# Genotypic Variation in Wheat Flour Lysophospholipids

**DOI:** 10.3390/molecules22060909

**Published:** 2017-05-31

**Authors:** Lei Liu, Qi Guo, Zhonghu He, Xianchun Xia, Daniel L. E. Waters, Carolyn A. Raymond, Graham J. King

**Affiliations:** 1 Southern Cross Plant Science, Southern Cross University, Lismore, NSW 2480, Australia; qi.guo.cn@hotmail.com (Q.G.); daniel.waters@scu.edu.au (D.L.E.W.); carolyn.raymond@scu.edu.au (C.A.R.); graham.king@scu.edu.au (G.J.K.); 2 Institute of Crop Science, Chinese Academy of Agricultural Sciences, Beijing 100081, China; xiaxianchun@caas.cn; 3 CIMMYT China Office, Chinese Academy of Agricultural Sciences, Beijing 100081, China

**Keywords:** wheat, lysophospholipid, kernel hardness, genotype

## Abstract

Lysophospholipids (LPLs) are the most abundant polar lipids in wheat endosperm and naturally complex with amylose, affecting starch physicochemical properties. We analyzed LPLs in wheat flour from 58 cultivars which differ by grain hardness using liquid chromatography mass spectrometry (LCMS). There were significant differences in LPL content between cultivars, demonstrating that genotype rather than environment contributes most to the total variance in wheat endosperm LPLs. Polar lipids such as LPLs may play a role in grain hardness through their interaction with puroindoline proteins, however, no strong correlation between kernel hardness and LPLs was detected. This may reflect the location of LPLs within the starch granule as opposed to the puroindoline proteins outside starch granules. LPLs may have an indirect relationship with kernel hardness as they could share the same origin as polar lipids that interact with puroindoline on the starch granule surface.

## 1. Introduction

Wheat is an important source of starch and protein for more than 35% of the world’s population, making it the single most important food crop produced worldwide [1]. Lysophospholipids (LPLs) are a major class of polar lipids that complex with amylose in cereal starch granules [2]. Although wheat endosperm polar lipids, including LPLs, are a minor fraction of the wheat flour by mass, they are important to bread making quality because they enhance loaf volume [3]. LPLs such as lysophosphatidylcholine (LPC) could complex with puroindoline during dough mixing and fermentation and synergistically enhance foam stabilization and bread crumb structure, an important character for bread quality [4]. However, most LPLs are starch lipids which are strongly bound in the starch granules and may be largely unavailable to affect dough processing before starch gelatinization occurs [5]. Nevertheless, LPLs complex with amylose and influence starch pasting and thermal properties [6], and such amylose-LPL complexes moderate starch digestion which may help in preventing metabolic disorders and colon cancer [7].

Soft wheats have high levels of polar lipids [8,9], suggesting that polar lipids may be associated with kernel hardness, a major quality trait that influences the wheat supply chain from farmers to consumers [4]. Polar lipids on the surface of water-washed wheat endosperm starch granules, mostly glyco- and phospholipids, display strong affinity for puroindolines and possibly provide lipid “bridges” between the starch granule surface and puroindolines [10]. It is thought that hardness is probably influenced by an interaction between starch granules and the lipid-binding puroindoline proteins [11] in the protein matrix [1,12] which prevents strong adhesion between starch and storage proteins, and thus results in softer kernels [8,13]. However, the detailed mechanism by which polar lipids contribute to kernel softness/hardness is not known. The extraction of polar lipids from the surface of wheat endosperm starch granules with organic or organic-aqueous solvents with differing polarity does not remove puroindolines from the starch surface [8,9], indicating that more than one mechanism is involved in puroindoline-lipid-starch interactions [1]. Although this may be due to the solubility of puroindolines in these solvents, it is possible that puroindolines bind with polar lipids which are not extracted by organic or organic-aqueous solvents alone.

The total LPL content of wheat flour is higher than the combined content of the two major classes of starch surface polar lipids, glycolipids and phospholipids [14]. LPLs form inclusion complexes with amylose in starch granules, making organic solvent extraction at room temperature very difficult. Compared with the classic “defatting” by organic solvents, efficient extraction of LPLs requires both adequate water and heat to swell the native starch granule, which then enables the lipids to be solubilized by alcohol [15]. LPLs are not thought to concentrate on the starch surface [2], however, LPLs bind puroindolines [4] and this may prevent them from being extracted by organic solvents.

The relative contribution of environmental and genetic factors to wheat grain LPL composition has yet to be determined. Thus, the aim of this research was to quantify LPL variation in wheat flour derived from 58 cultivars in order to assess the relative contribution of environmental and genetic factors in LPL composition.

## 2. Materials and Methods

### 2.1. Plant Materials

**Figure 1:**
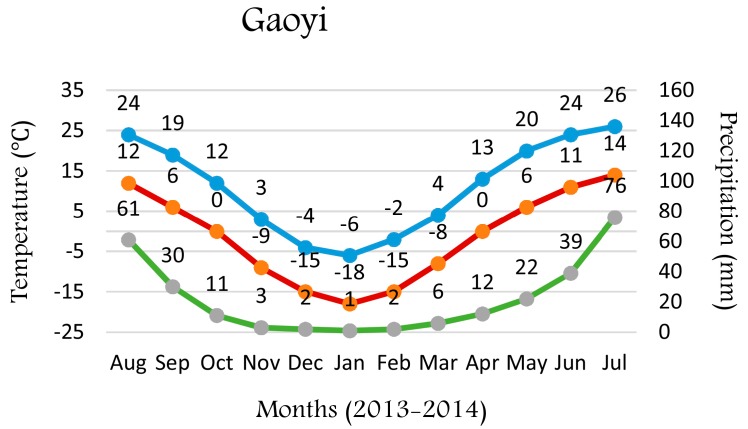

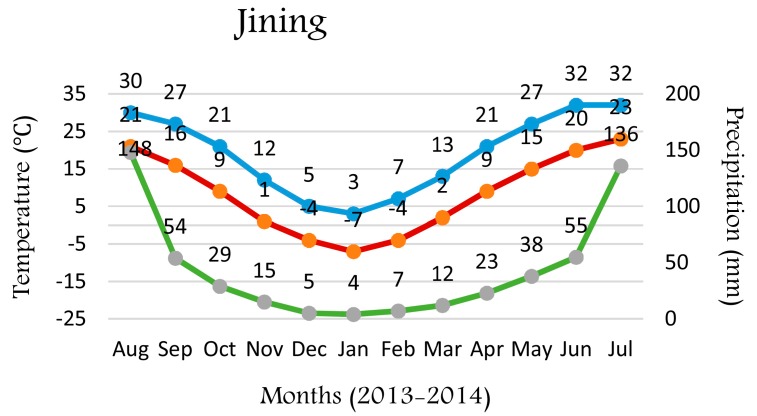
Precipitation and temperature of experiment stations. Cultivars were sown in October 2013 and harvested in June 2014 in both stations.

A total of 58 winter wheat cultivars and advanced lines were used in this study; two genotypes from USA, four from Australia, and 52 from the six major wheat producing provinces of China (Table S1 in supplementary materials). A randomized complete block design with two replications and plots consisting of 1.5 m rows, 20 cm apart, with 50 germinating seeds per plot, was used at two agricultural experiment stations; Jining (coordinates, 35.38, 116.59; Altitude: ~45 m, average precipitation 400–800 mm), Shandong Province, China and Gaoyi (coordinates, 37.62, 114.58; Altitude: ~53 m, average precipitation 400–800 mm) Hebei Province, China (see Figure 1 for average daily temperature), over the 2013–2014 growing season. All grain samples were cleaned after harvest and a falling number >300 s indicated that they were free from sprouting damage.

The samples were tempered overnight to 14.5%, 15.5%, and 16.5% moisture contents for soft, medium, and hard types, respectively. Milling was performed in a Brabender Quadrumat Junior laboratory mill based on the American Association of Cereal Chemists (AACC) method 26–50.01 to a flour extraction rate of 65%.

### 2.2. Hardness Measurement

Kernel hardness was determined using a Single Kernel Characterization System (SKCS) 4100, following the manufacturer’s instructions (Perten Instruments North America Inc. Springfield, IL, USA). Mean, standard deviation, and distribution of the SKCS hardness index were used to classify 300-kernel samples of each genotype, where an SKCS index of ≤40, 40–59, ≥60 determined a classification of soft, medium, and hard, respectively.

### 2.3. Chemicals

All organic solvents were HPLC or LCMS grade. The LPL standards, 1-oleoyl-2-hydroxy-*sn*-glycero-3-phosphoethanolamine (LPE 18:1), 1-palmitoyl-2-hydroxy-*sn*-glycero-3-phosphoethanol-amine (LPE 16:0), 1-oleoyl-2-hydroxy-*sn*-glycero-3-phosphocholine (LPC 18:1), 1-palmitoyl-2-hydroxy-*sn*-glycero-3-phosphocholine (LPC 16:0), were purchased from Avanti Polar Lipids Inc. (Alabaster, AL, USA).

### 2.4. Extraction of LPLs

The LPL extraction method of Liu et al. (2014) [16] was modified to increase sample throughput. Wheat flour (~15 mg) from each sample was placed in a 2-mL Agilent HPLC screw cap vial and freeze-dried. The flour was extracted with 75% *n*-propanol (*n*-propanol/water, 75/25, *v*/*v*; 0.8 mL) for 2 h at 100 °C in the same HPLC vial. The HPLC vials were weighed before and after heat extraction to ensure solvent loss did not affect the solvent/sample ratio (*w*/*w*). A 0.5-mL aliquot of the extract was transferred to another HPLC vial for the LCMS analyses. To check the difference between the total LPLs and the starch LPLs, some of wheat flour samples were also extracted by chloroform/methanol (2:1) [16] to remove the non-starch LPLs prior to the extraction by 75% n-propanol at 100 °C.

### 2.5. LCMS Analysis of LPLs

The LCMS method [16] was used for the analysis of wheat flour LPL extracts. Briefly, Agilent LCMS (HPLC1290 and Single Quadrupole MS 6120) equipped with an Agilent Eclipse Plus C18 RRHD column (1.8 μm; 50 × 2.1 mm internal diameter) and a linear gradient elution program was used for the analysis. The mobile phase containing MilliQ H_2_O and 0.005% trifluoroacetic acid (TFA) and acetonitrile with 0.005% TFA was programmed from 10% to 99% acetonitrile in 10 min with a flow rate of 0.3 mL/min and held at 99% acetonitrile for 1.5 min. The injection volume was set at 3 μL per injection. The electrospray ionization (ESI) and Single Ion Monitor (SIM) mode were set up to detect 10 ions simultaneously using four available mass selective detection (MSD) signal channels, as reported previously [16].

### 2.6. Statistical Analysis

Analyses of variance plus estimation of variance components and correlation coefficients were conducted using the Statistical Analysis System (SAS Institute Inc., version 9.2, 2012, Cary, NC, USA). Restricted maximum likelihood (REML) analyses and principal component analysis (PCA) were conducted using Genstat software (VSN International 2014). REML analyses partitioned the total variance to determine the percentage contribution from genotypes, environments (locations), replicates, and the G × E interaction.

Correlations (r) amongst the lysophospholipids and with hardness were tested for significance of difference from zero. Due to the large sample size (*n* > 50), relatively small *r* values were significant (0.27 for 5% and 0.35 for 1% for *n* = 50) but, whilst significant, such small values do not indicate a strong relationship. For this study, an *r* value of 0.7 or greater was considered a strong relationship.

PCA aimed to identify groupings of similar cultivars and the traits driving these groupings. The PCA was based on the correlation matrix to avoid biasing the results towards traits with high variance. PCA loadings and scores were extracted and the values for PCA components 1 vs. 2 were plotted. Hierarchical cluster analysis was carried out using the Ward Linkage method and squared Euclidean distance (IBM Corp. Released 2013. IBM SPSS Statistics for Windows, Version 22.0. Armonk, NY, USA).

## 3. Results

### 3.1. Concentration of Individual and Total Lysophospholipids

The major lysophosphatidylcholines (LPCs) were LPC18:2 and LPC16:0, and the major lysophosphatidylethanolamines (LPEs) were LPE18:2 and LPE16:0 (Table 1). The concentrations of major LPLs (LPC18:2, LPC16:0, and LPE18:2) had a relatively small coefficient of variation (CV) (8.3%, 7.4%, and 8.6%), while the concentrations of minor LPLs (LPC14:0, LPE 18:3, and LPE18:1) had relatively large coefficients of variation (CV) (60.2%, 44.6%, and 29.3%) (Table 1). LPE14:0 was below the detection limit (signal/noise = 3). The concentration of total LPCs was higher than total LPEs and accounted for nearly 90% of the total LPLs (Table 1). The LCMS analysis shows that the difference between the amount of total LPLs and starch LPLs was not obvious (Figure S1) as reported by Hargin and Morrison [2].

### 3.2. Genetic and Environmental Effects on Wheat LPL Composition

Total LPLs varied significantly among the 58 cultivars (ANOVA; *p* < 0.01), with genotype accounting for 49.9% of the total variation (Table 2 and Table 3). Environment, genotype × environment interaction, and biological replicate were not significant (*p* > 0.05) (Table 2 and Table 3). Similarly, the effect of genotype on total LPCs and total LPEs was significant (*p* < 0.01) and accounted for 49.1% and 58.5% of the total variance, respectively, whereas environment, genotype × environment interaction, and biological replicate were not significant (*p* > 0.05) (Table 2). Although LPL composition varied significantly among cultivars (*p* < 0.01), the genotype variance of LPC14:0 and LPE18:1 only accounted for 35.4% and 35.8% of total variance, while the genotype variance of LPC18:3, LPE18:3, and LPE16:0 accounted for 70.1%, 74.5%, and 66.8%, respectively (Table 2 and Table 3). Compared with major LPCs or LPEs (LPC18:2, LPC16:0, LPE18:2, and LPE16:0), environment accounted for more of the total variance of some minor LPCs and LPEs (LPC18:1, LPC14:0 and LPE18:1), representing a larger and more significant effect (*p* < 0.05 or *p* < 0.01) (Table 2 and Table 3).

### 3.3. Correlation Analysis of Lysophospholipids

The correlation between LPC and LPE components with the same fatty acid composition, LPC18:3 and LPE18:3 (*r* = 0.90, *p* < 0.001), LPC18:1 and LPE18:1 (*r* = 0.82, *p* < 0.001) for example, were higher than those with different fatty acid compositions (*r* < 0.69) (Figure S2). The correlation between minor LPC and LPE components with the same fatty acid composition, (LPC18:3 and LPE18:3, LPC18:1 and LPE18:1) was relatively higher (*r* > 0.82) than the major LPCs and LPEs with the same fatty acid composition (LPC18:2 and LPE18:2, LPC16:0 and LPE16:0) (*r* < 0.73) (Figure S2). LPL composition between locations was significantly correlated (*p* < 0.001), suggesting the environmental effect on wheat LPL composition was limited. Most of the minor LPLs (LPC18:3, LPC18:1, LPC14:0, LPE18:3, LPE18:1, LPE16:0) had relatively higher correlation coefficients between locations (*r* = 0.82, 0.76, 0.74, 0.87, 0.69, 0.80) than major LPL compositions (LPC18:2, LPC16:0, LPE18:2, *r* = 0.69, 0.67, 0.66).

### 3.4. Principal Component Analysis and Hierarchical Cluster Analysis of Lysophospholipids

**Figure 2:**
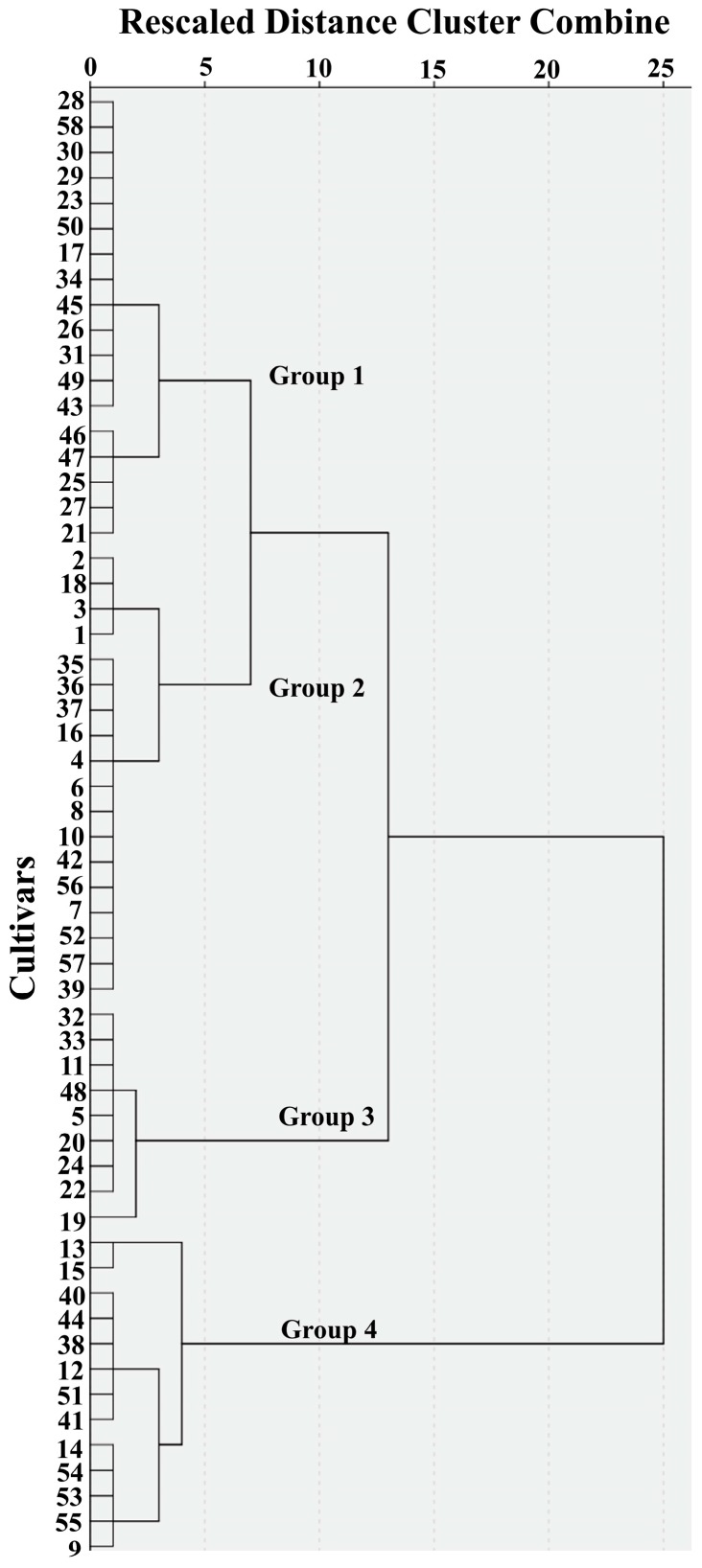
Hierarchical cluster analysis dendrogram (Ward’s method) based on individual LPL content of 58 wheat cultivars.

**Figure 3:**
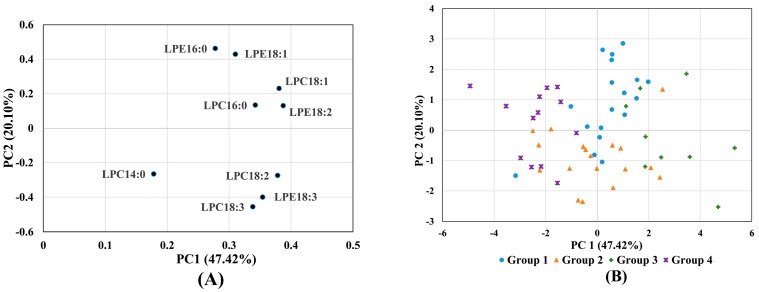
Results from principal components analysis (PCA) of LPL data with (**A**) loadings for each LPL and (**B**) scores for each cultivar plotted for the first two PCA components.

Hierarchical cluster analysis of the 58 cultivars based on individual LPLs identified three groups at a relative distance of 13 and four groups at a relative distance of 7 (Figure 2). Hierarchical cluster analysis of LPL data grouped the samples in part by origin; Shandong province genotypes were only found in Group 1, Group 2 was dominated (12 of 18) by genotypes from Henan province, while Group 4 was dominated (7 of 13) by Beijing genotypes (Table S1). The first two PCA components explained 47.4% and 20.1% of the variation, respectively, with LPC14:0 appearing to be the main discriminator on component 1 (Figure 3A). For component 2, LPE 16:0 and LPE18:1 were positively loaded whilst LPC18:3 and LPE18:3 were negatively loaded. Figure 3B indicated no obvious pattern of groupings of cultivars, even though some trends were observed when plotting the four cluster analysis groups in four different colors.

### 3.5. Correlation Analysis of Lysophospholipids and Kernel Hardness

Most LPLs had a positive relationship with kernel hardness except LPC 14:0, although no strong correlation was observed between kernel hardness and the LPLs when analyzing all 58 samples together (Table 4 and Figure S3). The correlation analysis of samples grouped by breeding location (Beijing, Shandong, Henan, number of cultivars > 6) (Table S1) or the cluster analysis (four groups) (Figure 2) found relatively strong correlations (*r* >0.7 or ≤ −0.7) for LPE 16:0 within the cluster analysis Group 3 (*r* = 0.70) and LPC 18:1 within the Shandong breeding program (*r* = −0.86) (Table S2).

## 4. Discussion

Although LPLs are the major class of wheat endosperm lipids, the endosperm LPL composition of a very small number (11) of cultivars has been investigated and compared [17]. The range of total endosperm LPLs in the 58 wheat cultivars reported here (7174–9811 μg/g dry wt) are within the range of the previous report (7290–10,470 μg/g dry wt) [17]. LPLs are present in the starch of many cereal grains [17], and although the total endosperm LPLs found in wheat cultivars are in a similar range to those found in rice [18], there were fewer total LPEs in wheat (~12% of total LPLs) than in rice endosperm (~18% of total LPLs). 

The proportion of major LPLs with unsaturated fatty acids were higher in wheat cultivars (e.g., LPC 18:2 and LPE 18:2, 43.3–52.2%, Table 1) than in rice cultivars (e.g., LPC 18:2 and LPE 18:2, 19.3–33.3%) [16,17], a difference that may reflect the evolutionary or domestication history of each species. Unsaturated fatty acids maintain fluidity and therefore functionality at lower temperatures than saturated fatty acids [19]. Rice is a tropically adapted species that evolved in South and South East Asia [20], while the progenitors of wheat evolved in the eastern Mediterranean [21], a region which currently has a lower mean annual temperature than South and South East Asia. As each of these species evolved, the lower temperatures experienced by wheat relative to rice may have resulted in a higher proportion of unsaturated fatty acids being needed in the major wheat LPLs in order to maintain equivalent levels of lipid functionality. Alternatively, these differences in lysophospholipid ratios may arise from human selection for traits which are in part due to lysophospholipid content and or composition.

In line with rice [18], genotype is the greatest contributor to total variance in wheat endosperm LPLs, suggesting that there is scope to breed wheat cultivars with desired endosperm LPL composition and content. Although endosperm LPLs differed among wheat cultivars, the influence of environment was not exhaustively investigated. A recent study indicated that environmental conditions during growth may have a significant effect on the properties of amylose-lipid complexes and starch properties in wheat endosperm [22], which may be due to unidentified environmental effects on LPLs, the major lipids complexed with amylose in wheat endosperm starch. However, the contribution of environment to the total variance in the current study was minimal for the total and major individual LPLs, suggesting the effect of environment on wheat endosperm LPLs is limited. The samples used by Kwasniewska-Karolak et al. [22] consisted of three genotypes only and were grown in three consecutive years at one location while our samples were grown in two locations within one year, and although there were substantial temperature differences between the two locations (Figure 1), these differences did not cause a significant genotype by environment interaction.

The precursors of LPC and LPE in wheat endosperm are not entirely clear, however, they are most likely hydrolyzed from diacyl phosphatidylcholine (DPC) and diacyl phosphatidylethanol-amine (DPE) by phospholipase A_2_ (PLA2) or phospholipase A1 (PLA1), as has been demonstrated in other plant tissues [23,24]. Therefore, the strong correlation between the LPC and LPE with the same fatty acid composition, which is similar to that found in rice [18], may be inherited from their precursors DPC and DPE. In rice, the fatty acid composition of both LPC/LPE and DPC/DPE are correlated [18,25], however, to the best of our knowledge, the correlations of fatty acid composition in DPC and DPE have not been reported in wheat grain before. 

The concentration of palmitic acid (37~47%, Table 1) was higher in LPL than reported in other lipid fractions in wheat grain such as non-starch phospholipids (23~27%) [26]. This may confirm that LPC and LPE are more likely the hydrolyzed products of DPC and DPE by PLA2 since the fatty acid in the *sn*-1 position is less unsaturated (e.g., palmitic acid) than those in the *sn*-2 position (e.g., linoleic acid) and PLA2 can remove the acyl chain at the position *sn*-2 (e.g., linoleic acid) of DPC or DPE to form LPC or LPE [24,27]. Transcriptome analysis of cereal grain filling with special consideration of genes related to PLA2 may shed some light on the mechanisms of cereal endosperm LPL biosynthesis.

Inbred crops, such as wheat, are sensitive to environment which makes it necessary to breed each genotype for a narrow environmental range [28], hence the need for provincial breeding programs. Breeders commonly use at least one parental genotype that is adapted to the target environment in their crosses, and this will be reflected in the genotype of the cultivars which arise from each provincial breeding program. Our results suggest that LPL composition in these samples is determined largely by genotype, with environment playing a minor role. Groupings (Figure 2) based on LPL composition therefore reflect underlying genetic differences between breeding programs (Table S1), differences which may have arisen due to a number possible factors. Although LPL composition in these samples is determined largely by genotype, environment may have played a role during the selection of these genotypes. It is possible that LPL composition may determine an environmentally dependent functional trait, germination, or maturation rate for example, and alleles which influence LPL composition may have been selected within the environment of each breeding program because of this functional significance. Equally, alleles which influence LPL composition may have been selected because they are linked to genes of importance within each program. It is possible then that serendipitous selection of alleles that influence LPL composition may have taken place and LPL composition simply reflects the pedigree of the genotypes which dominate the genetic background of each provincial breeding program. Further genome analysis, including analysis of parental lines, would assist in answering which of the forces of selection that operate in these breeding programs has given rise to these differences in LPL composition.

Finnie et al. [29] studied variation in polar lipid composition among near-isogenic wheat lines possessing different puroindoline haplotypes and postulated a mechanism of wheat endosperm hardness which included a minimum four-way interaction between the starch granule surface, storage proteins, puroindoline proteins, and polar lipids on the starch granule surface. LPLs are the major endosperm polar lipid, and the current research demonstrates there is no strong correlation between wheat kernel hardness and LPLs. This may be because LPLs are located within the starch granule and have little opportunity to interact with puroindolines located outside the starch granule, although puroindolines are lipid-binding proteins that could bind with LPLs [30]. In the current study, there was a positive relationship between kernel hardness and LPLs while others have reported a negative relationship between kernel hardness and polar lipids on the starch granule surface [8]. A number of researchers hypothesized or were inclined to think that puroindolines may stabilize polar lipids that are the remnants of the amyloplast and prevent them from breakdown during seed maturation and desiccation [1,31]. The weak positive relationship between kernel hardness and total LPLs found here suggests that this may be the case, since harder kernel could be the result of less puroindolines which also could cause more degradation of polar lipids of amyloplast membrane to LPLs.

## 5. Conclusions

Endosperm LPLs in wheat are similar to those in rice, although the LPC/LPE ratio and the level of unsaturated fatty acid are higher in wheat LPLs. Genotype contributes the most to the total variance of wheat endosperm LPLs, suggesting that it is possible to breed wheat grain for high or low LPLs. There were no strong correlations between LPLs and wheat kernel hardness, probably due to the lack of physical contact between the LPLs and puroindoline protein.

## Figures and Tables

**Table 1:** Individual and total LPL concentration in freeze-dried wheat flour ^1^.

LPLs	Mean (μg/g)	±SD ^2^ (μg/g)	CV ^3^ (%)	Percentage ^4^ (%)
LPC 18:3	246.9	±43.8	17.7	2.0–3.8
LPC 18:2	3578.8	±298.5	8.3	38.0–44.8
LPC 18:1	587.2	±75.3	12.8	4.9–8.3
LPC 14:0	13.4	±8.1	60.2	0–0.5
LPC 16:0	3195.3	±236.7	7.4	33.3–41.4
LPE 18:3	14	±6.3	44.6	0–0.3
LPE 18:2	552.2	±47.7	8.6	5.3–7.4
LPE 18:1	29.5	±8.7	29.3	0.1–0.6
LPE 16:0	379.7	±46.1	12.1	3.3–5.5
Total LPCs	7621.5	±553.9	7.3	87.0–90.1
Total LPEs	975.4	±90.0	9.2	9.9–13.0
Total LPLs	8596.9	±620.3	7.2	100

^1^ Results of 58 cultivars × 2 locations × 2 biological replicates; ^2^ SD: standard deviation; ^3^ CV: coefficient of variation; ^4^ Percentage of individual LPLs in total LPLs.

**Table 2:** Variance analysis of wheat LPLs of 58 cultivars in two locations.

LPLs	Mean Square Values from Analysis of Variance (ANOVA)			
	Environment (Location)	Biological Replicate	Genotype	G × E ^2^
df ^1^	1	1	57	57
LPC 18:3	4785.2 **	35.1	6093.7 **	621.2
LPC 18:2	127,610	46,065.3	230,811 **	42,794.1
LPC 18:1	136,571 **	1879	15,433.3 **	2204.1
LPC 14:0	3489.8 **	5.1	135.7 **	22.1
LPC 16:0	100,213 *	30,206.3	148,042 **	30,041.8
LPE 18:3	37.8 *	21	127.7 **	9.1
LPE 18:2	303.4	320.5	6059.8 **	1109.8
LPE 18:1	4558.3 **	0.8	169.7 **	36.4 **
LPE 16:0	3.6	362.9	6441.7 **	734.6
Total LPCs	97,405.9	192,260	778,837 **	174,199.1
Total LPEs	6516.1	1779.2	22,645.9 **	3473
Total LPLs	53,541.5	230,966	980,810 **	212,343.2

^1^ degree of freedom; ^2^ genotype × environment interaction; * Significant at *p* < 0.05; ** significant at *p* < 0.01.

**Table 3:** Estimated percentage contribution of genotype, environment, and genotype × environment to total variance using restricted maximum likelihood (REML) approach.

LPLs	Environment (Location) %	Replicate %	Genotype %	G × E ^1^ %	Residual %
LPC 18:3	1.9	0	70.1	3.8	24.1
LPC 18:2	0.4	0.9	52.2	1.3	45.2
LPC 18:1	18.5	0	53.1	6.9	21.5
LPC 14:0	37.3	0	35.4	0.2	27.1
LPC 16:0	1.1	0	52.1	6.4	40.3
LPE 18:3	0.7	0	74.5	0	24.8
LPE 18:2	0	0	54.2	2.2	43.6
LPE 18:1	41.4	0	35.8	15.7	7.1
LPE 16:0	0	0	66.8	0.8	32.4
Total LPCs	0	0	49.1	5.4	45.4
Total LPEs	0.3	0	58.8	1.9	38.9
Total LPLs	0	0	49.9	4.2	45.9

^1^ G × E: Genotype × Environment.

**Table 4:** Correlation analysis of wheat flour lysophospholipids and wheat kernel hardness (SKCS *).

Hardness (*n* = 58)		**LPC18:3**	**LPC18:2**	**LPC18:1**	**LPC14:0**	**LPC16:0**	**LPE18:3**	**LPE18:2**	**LPE18:1**	**LPE16:0**	**Total LPC**	**Total LPE**	**Total LPL**
	*r* value	0.14	0.08	0.39	−0.10	0.32	0.15	0.18	0.34	0.39	0.25	0.35	0.28
	*p* value	0.306	0.530	0.002	0.443	0.013	0.253	0.173	0.008	0.002	0.055	0.007	0.034

* SKCS, single kernel characterization system.

## Supplementary Materials

Supplementary materials are available online.
