# Supplementary material for: Genotypic Variation in Wheat Flour Lysophospholipids
Source: Molecules. 2017 May 31;22(6):909. doi: 10.3390/molecules22060909 (PMC6152675; doi:10.3390/molecules22060909)
Supplement: Supplementary file 1 [file molecules-22-00909-s001.pdf]

Table S1. Information for the cultivars used in this research

ID	Name	Combination (Parents)	Location of Breeding Program	Breeding Institute
1	08CA255(13CA66)	Wenmai 6 x Sunstate	Henan	ICS, CAAS
2	12A25	Jimai 22 x Jishi 02-1	Henan	ICS, CAAS
3	12A29	Jimai 22 x Jishi 02-1	Henan	ICS, CAAS
4	12CA39	Zhengmai 366 x Yannong 19	Henan	ICS, CAAS
5	12pin404	954072 x Jinan 17	Henan	ICS, CAAS
6	13A38	Jishi 02-1 x Zhoumai 16	Henan	ICS, CAAS
7	13A39	Jishi 02-1 x Zhoumai 16	Henan	ICS, CAAS
8	13A47	Jishi 02-1 x Zhoumai 16	Henan	ICS, CAAS
9	13A48	Jishi 02-1 x Zhoumai 16	Henan	ICS, CAAS
10	Baxter	((Inia-66 x Gamut) x Cook) x (Jupateco x ((Lerma-rojo-64 x Sonora-64-A) x (SIB)Timgalen)	Australia	N/A
11	CA0493	CA9640 x Zhongyou 9507	Beijing	ICS, CAAS
12	CA0996	Yumai 34 x Lunxuan 9873	Beijing	ICS, CAAS
13	CA0998	Yumai 34 x Lunxuan 9873	Beijing	ICS, CAAS
14	CA1004	(Zhongyou 9507 x CA9640)-4/(30095 x Zhongyou 9701)	Beijing	ICS, CAAS
15	CA1062	Yumai 34 x Lunxuan 9873	Beijing	ICS, CAAS
16	ELLISON	N/A	Australia	N/A
17	GY12014	Jimai 22 x Jishi 02-1	Henan	ICS, CAAS
18	GY12023	Jimai 22 x Jishi 02-1	Henan	ICS, CAAS
19	Jagger	KS-82-W-418 x Stephens	USA	N/A
20	Karl	Plainsman-V x ((Kaw x Atlas-50) x (Parker*5 x Agent))	USA	N/A
21	LIVINGSTON	N/A	Australia	N/A
22	SUNZELL	N/A	Australia	N/A
23	Fengdecunmai 5	Zhoumai 16 x Zhengmai 366	Henan	ZFSI Co., LTD
24	Gaoyou 2018	9411 x 98172	Hebei	GCARI

25	Gaoyou 8901	77546-2 x Linzhangmai	Hebei	GCARI
26	Jimai 086029	Gaocheng 9411 x 200040919	Shandong	CRI, SDAAS
27	Jimai 20	Lumai 14 x 884187	Shandong	CRI, SDAAS
28	Jimai 22	935024 x 935106	Shandong	CRI, SDAAS
29	Jimai 23	Jimai 22x Yumai 34*3	Shandong	CRI, SDAAS
30	Jimai 24	Jimai 22 x Yumai 34*3	Shandong	CRI, SDAAS
31	Jinai 17	Linfen 5064 x Lumai 13	Shandong	CRI, SDAAS
32	Nongda 3615	Linfen 3118 x Nongda 3134	Beijing	CAU
33	Nongda 3753	Jingdong 8 x Heixiaomai 76	Beijing	CAU
34	Nongda 5363	Nongda 195 x Jing 98—270	Beijing	CAU
35	Shannong 11-28	N/A	Shaanxi	SDAU
36	Shan627	88119-19-3-5-10 x WX8911	Shaanxi	CANAFU
37	Shiluan 02-1	9411 x 9430	Hebei	HBNU
38	Shi 4185	Zhi 8094 x Baofeng 7228 x Shi 84-7120	Hebei	SJZARI
39	Shiyou 17	Ji935-352 x Lumai 21	Hebei	SJZARI
40	Shiyou 20	Ji 935-352 x Jinan 17	Hebei	SJZARI
41	Shunmai 1718	32S x Gabo	Shanxi	CRI, SXAAS
42	Wunong 986	Shan 253 x (97)107	Henan	YVTC
43	Xinong 509	VP145 x 86585	Shaanxi	CANAFU
44	Xinong 979	Xinong 2611 x (918 x 95 xuan 1)F1	Shaanxi	CANAFU
45	Xinmai 0208	Xinmai 18 x Shanmai 225	Henan	XXAAS
46	Xinmai 26	Xin 9408E1 x Jinan 17	Henan	XXAAS
47	Yumai 34	Aifeng 3 x ((Mengxian 201 × Niuzhute) x Yumai 2)	Henan	ZZARI
48	Zheng 5373	Aikang 58 x Xinong 979	Henan	HNAAS
49	Zhengmai 129	Zhengmai 366 x Liangxing 99	Henan	HNAAS
50	Zhengmai 366	Yumai 47 x PH82-2-2	Henan	HNAAS
51	Zhongmai 629	30095 x Zhongyou 9701	Beijing	ICS, CAAS
52	Zhongmai 895	Zhoumai 16 x Liken 4	Henan	ICS, CAAS
53	Zhongyou 206	CA9614 x Zhongyou 9507	Beijing	ICS, CAAS
54	Zhongyou 255	Wenmai 6 x Sunstate	Beijing	ICS, CAAS

55	Zhoumai 24	Zhoumai 16 x Shanyou 225	Henan	ZKAAS
56	Zhoumai 26	Zhoumai 24 x Zhoumai 22	Henan	ZKAAS
57	Zhoumai 32	Aikang 58 x Zhoumai 24	Henan	ZKAAS
58	Zhouyuan 9369	PH82-2-2 x 866-34	Shandong	SZSI Co., LTD

CANAFU: College of Agronomy, Northwest A&F University;
CAU: China Agricultural University;
CRI, SDAAS: Crop Research Institute, Shandong Academy of Agricultural Sciences;
CRI, SXAAS: Cotton Research Institute, Shanxi Academy of Agricultural Sciences;
GCARI: Gaocheng Agricultural Research Institutes;
HBNU: Hebei Normal University;
HNAAS: Henan Academy of Agricultural Sciences;
ICS, CAAS: Institute of Crop Sciences of Chinese Academy of Agricultural Sciences;
SDAU: Shandong Agricultural University;
SJZARI: Shijiazhuang Agricultural Research Institutes, Hebei;
SZSI Co., LTD: Shandong Zhouyuan Seed Industry Co., LTD;
XXAAS: Xinxiang Academy of Agricultural Sciences, Henan;
YVTC: Yangling Vocational&Technical College;
ZFSI Co., LTD: Zhengzhou Fengdekang Seed Industry Co., LTD;
ZKAAS: Zhoukou Academy of Agricultural Sciences;
ZZARI: Zhengzhou Agricultural Research Institutes.

Table S2. Correlation analysis of wheat flour lysophospholipids and wheat kernel hardness by groups

Groups		LPC 18:3	LPC 18:2	LPC 18:1	LPC 14:0	LPC 16:0	LPE 18:3	LPE 18:2	LPE 18:1	LPE 16:0	Total LPCs	Total LPEs	Total LPLs
Group 1	r	-0.315	0.344	0.396	-0.678	0.04	-0.256	0.391	0.361	0.224	0.223	0.319	0.285
(n=18)	P	0.202	0.162	0.104	0.002	0.875	0.306	0.109	0.141	0.372	0.374	0.198	0.251
Group 2	r	0.609	0.368	0.488	-0.002	0.312	0.565	-0.055	0.368	-0.146	0.607	0.011	0.527
(n=18)	P	0.007	0.133	0.04	0.995	0.208	0.015	0.829	0.133	0.562	0.008	0.964	0.025
Group 3	r	0.147	0.409	-0.053	-0.074	0.354	0.092	0.019	-0.313	0.694	0.383	0.325	0.448
(n=9)	P	0.705	0.275	0.893	0.85	0.351	0.814	0.961	0.412	0.038	0.309	0.394	0.227
Group 4	r	0.094	-0.288	0.454	0.015	0.298	0.144	0.319	0.297	0.466	-0.004	0.476	0.059
(n=13)	P	0.76	0.34	0.119	0.963	0.322	0.639	0.289	0.324	0.109	0.99	0.1	0.848
Henan	r	0.42	0.16	0.468	-0.131	0.353	0.497	0.295	0.5	0.221	0.504	0.394	0.496
(n=23)	P	0.046	0.467	0.024	0.55	0.098	0.016	0.172	0.015	0.312	0.014	0.063	0.016
Shangdong	r	0.143	-0.162	-0.861	-0.661	-0.564	0.321	-0.619	-0.128	-0.491	-0.439	-0.621	-0.555
(n=7)	P	0.759	0.728	0.013	0.106	0.187	0.483	0.138	0.785	0.263	0.324	0.137	0.196
Beijing	r	-0.157	-0.373	-0.062	-0.575	-0.481	-0.329	-0.224	0.104	-0.418	-0.393	-0.302	-0.389
(n=11)	P	0.646	0.258	0.857	0.064	0.134	0.324	0.508	0.762	0.201	0.232	0.367	0.237

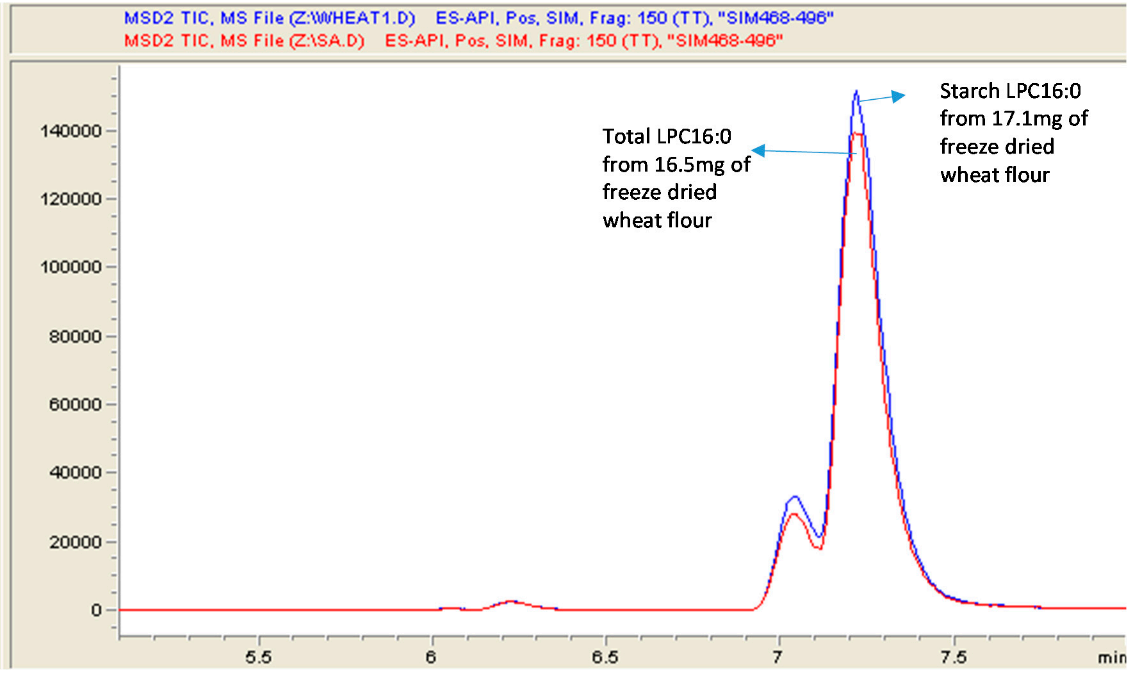

Figure S1. LCMS chromatograms of starch and total LPC16:0 of same wheat flour sample.

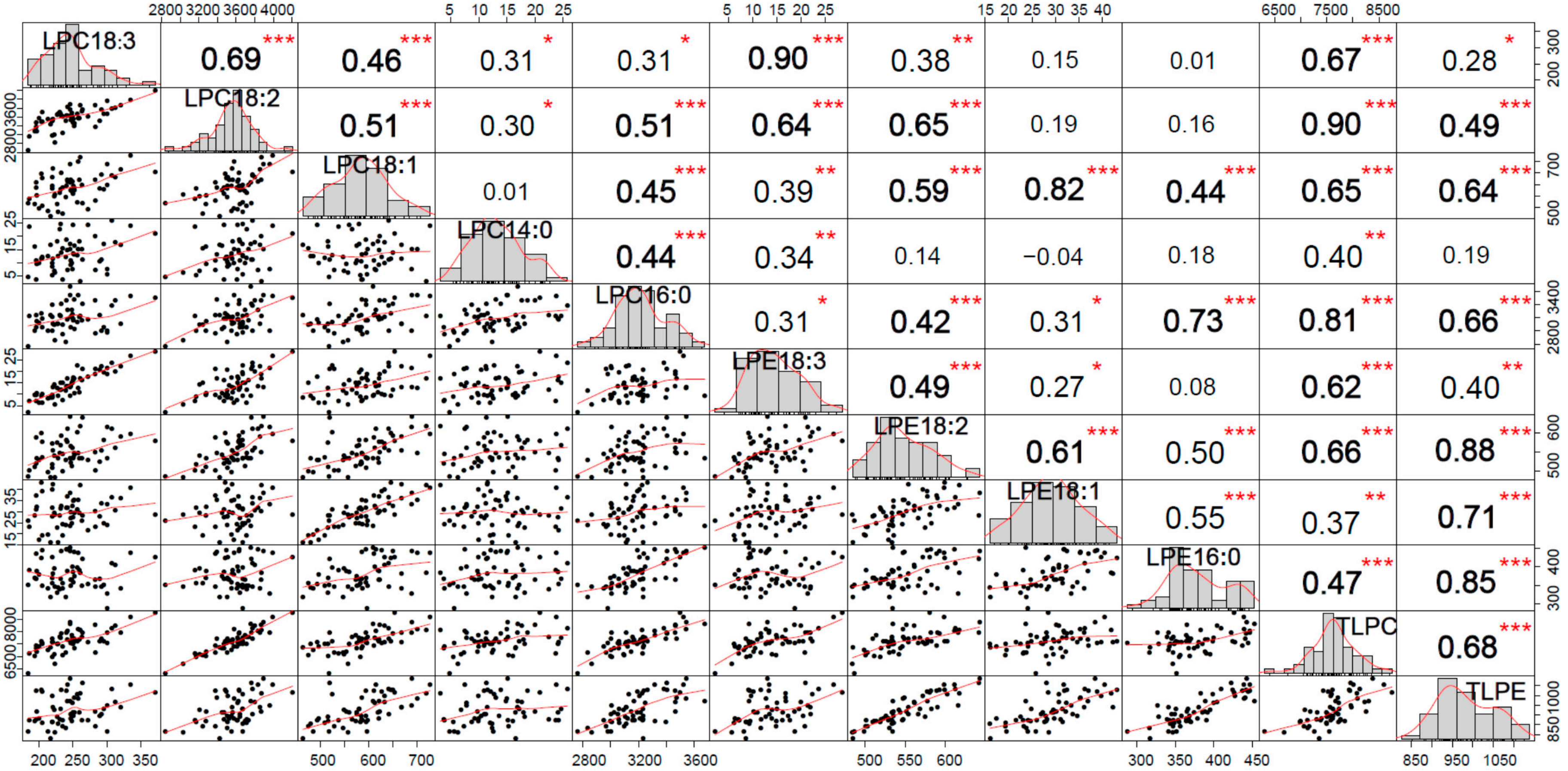

Figure S2. Correlation analysis of wheat lysophospholipids

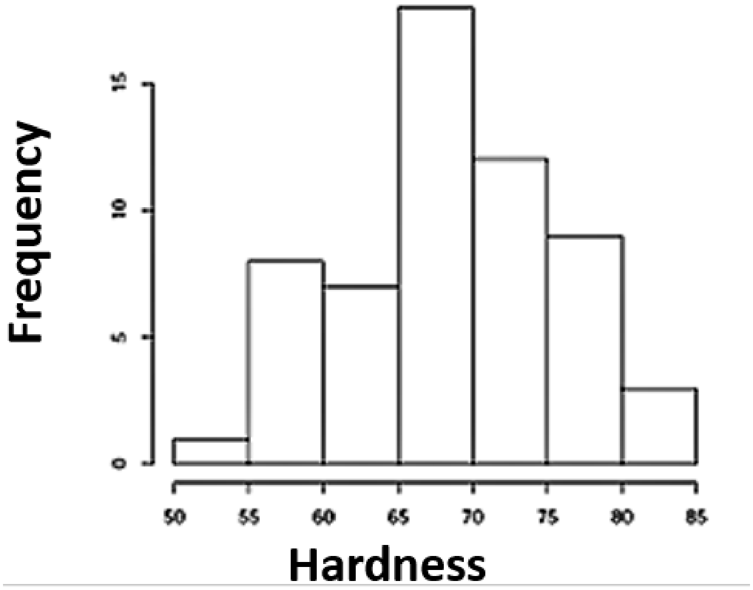

Figure S3. Histogram of wheat kernel hardness
